# Divergent natural selection alters male sperm competition success in *Drosophila melanogaster*

**DOI:** 10.1002/ece3.8567

**Published:** 2022-02-16

**Authors:** Ralph Dobler, Marc Charette, Katrin Kaplan, Biz R. Turnell, Klaus Reinhardt

**Affiliations:** ^1^ Animal Evolutionary Ecology Institute of Evolution and Ecology Eberhard Karls University of Tubingen Tübingen Germany; ^2^ 9169 Applied Zoology Institute of Zoology Technische Universität Dresden Dresden Germany; ^3^ 6363 Department of Biology University of Ottawa Ottawa Ontario Canada

**Keywords:** experimental evolution, hypercapnia, hypoxia, natural selection, sexual selection, sperm competition

## Abstract

Sexually selected traits may also be subject to non‐sexual selection. If optimal trait values depend on environmental conditions, then “narrow sense” (i.e., non‐sexual) natural selection can lead to local adaptation, with fitness in a certain environment being highest among individuals selected under that environment. Such adaptation can, in turn, drive ecological speciation via sexual selection. To date, most research on the effect of narrow‐sense natural selection on sexually selected traits has focused on precopulatory measures like mating success. However, postcopulatory traits, such as sperm function, can also be under non‐sexual selection, and have the potential to contribute to population divergence between different environments. Here, we investigate the effects of narrow‐sense natural selection on male postcopulatory success in *Drosophila melanogaster*. We chose two extreme environments, low oxygen (10%, hypoxic) or high CO_2_ (5%, hypercapnic) to detect small effects. We measured the sperm defensive (P1) and offensive (P2) capabilities of selected and control males in the corresponding selection environment and under control conditions. Overall, selection under hypoxia decreased both P1 and P2, while selection under hypercapnia had no effect. Surprisingly, P1 for both selected and control males was higher under both ambient hypoxia and ambient hypercapnia, compared to control conditions, while P2 was lower under hypoxia. We found limited evidence for local adaptation: the positive environmental effect of hypoxia on P1 was greater in hypoxia‐selected males than in controls. We discuss the implications of our findings for the evolution of postcopulatory traits in response to non‐sexual and sexual selection.

## Graphical abstract

Natural selection can impact sexual traits, including postcopulatory traits, potentially driving ecological speciation via sexual selection. Here, we show that sperm competitive success, both defensive (P1) and offensive (P2), decline in *D. melanogaster* in response to selection under low oxygen (hypoxia), but not high CO2 (hypercapnia). We conclude that natural selection can shape postcopulatory traits and may thereby drive population divergence.

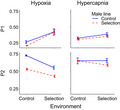

## 1 INTRODUCTION

Sperm competition occurs when the ejaculates of two or more males compete for the fertilization of a female's eggs (Parker, 1970; Simmons & Wedell, 2020). The sperm and ejaculate traits underlying competitive fertilization success are shaped by postcopulatory sexual selection (Parker, 2020). Environmental factors can also affect the outcome of sperm competition, both in the short term via plastic responses (De Nardo et al., 2021; Dobler & Reinhardt, 2016; Vasudeva et al., 2019) and over multiple generations via natural selection (Singh et al., 2016). Note that for the purpose of this paper, we will use the term “natural selection” as a shorthand for “narrow‐sense” natural selection (Endler, 1986; Shuker & Kvarnemo, 2021), that is, non‐sexual selection. Sexual selection, by contrast, is any selection due to non‐random success in the competition for access to gametes (Andersson, 1994; Shuker & Kvarnemo, 2021).

The relationship between sexual and natural selection on male reproductive traits is complex. Natural selection on these traits, or on traits with which they are genetically correlated through pleiotropy or linkage, may work in concert with sexual selection or may drive them away from their optimal sexually selected values (Fricke et al., 2010; House et al., 2013; Padró et al., 2019; Sharma et al., 2012). If these sexually selected optima are environmentally dependent, then selection can lead to local adaptation of reproductive traits, whereby males selected in a given environment outperform other males when competition occurs in that environment (Kawecki & Ebert, 2004). Such local adaptation can ultimately lead to reproductive isolation between ecologically divergent populations and to speciation by sexual selection (Rundle & Nosil, 2005).

An increasing number of studies have investigated the effects of divergent natural selection on male reproduction, with many of them focusing on local adaptation as measured by mating success. In *Drosophila melanogaster*, males selected under elevated temperatures showed higher mating success in the selection environment than did control males in one study (Dolgin et al., 2006), though not in another (Correia et al., 2010). Similarly, *D*. *melanogaster* males selected for cold shock resistance (Singh et al., 2015, 2016) and *D*. *buzzatii* males selected for heat shock resistance (Sambucetti & Norry, 2015) had higher mating success than controls when both were exposed to the relevant stressor. The same pattern was shown in both *D*. *melanogaster* (Gefen & Gibbs, 2009) and the mosquito *Anastrepha ludens* (Tejeda et al., 2017) in response to selection for desiccation resistance. However, *D*. *melanogaster* males selected under increased larval densities (Shenoi & Prasad, 2016) or dietary cadmium levels (Arbuthnott & Rundle, 2014) had no mating advantage over control males in the selection environment.

In contrast to this literature on male mating success, little work has been done on how sperm competitiveness responds to selection under different environmental conditions. One experiment, in *D*. *melanogaster*, found that selection for over 4 years under diets containing either ethanol or cadmium affected sexual conflict traits like male harm and female resistance (Arbuthnott et al., 2014), but not the outcome of sperm competition, as measured by the proportion of offspring fertilized by the first or second male to mate with a female (P1 and P2) (Arbuthnott et al., 2014).

Even less research exists directly testing whether divergent natural selection can lead to local adaptation in the postcopulatory arena, such that the sperm of selected males outcompetes that of control males in the selection environment. Certainly, a number of studies have examined how sperm traits respond plastically to environmental variables like diet (Engqvist, 2008), rearing density (Morrow et al., 2008), and, especially, temperature (Adriaenssens et al., 2012; Blanckenhorn & Hellriegel, 2002; Fenkes et al., 2017; Gasparini et al., 2018; Iglesias‐Carrasco et al., 2020; Kekäläinen et al., 2018; Vasudeva et al., 2019). Several others have investigated environmental effects on sperm competitiveness *per se* (diet: Almbro et al., 2011; De Nardo et al., 2021; Rahman et al., 2014); rearing density: (Amitin & Pitnick, 2007); temperature: (van Lieshout et al., 2013; Sales et al., 2018; Vasudeva et al., 2014); elevated CO_2_ (hypercapnia) (Dobler & Reinhardt, 2016)).

As far as we are aware, however, only two studies have compared sperm traits or competitiveness across both immediate environments and artificial selection histories. One, in *D*. *melanogaster*, found local adaptation by males selected for cold shock resistance, whereby selected males had higher sperm offensive ability (P2) than did control males after cold shock exposure (Singh et al., 2016). The other, in guppies (*Poecilia reticulata*), found a negative immediate effect of rearing temperature on sperm length but a positive effect over the course of selection, with warm‐adapted males producing longer sperm than control males in both temperature environments (Breckels & Neff, 2014). A third study, comparing sperm motility in natural rather than experimentally selected populations of *D*. *subobscura*, likewise found a negative immediate effect of rearing temperature but no evidence of local adaptation, as southern males had higher motility than northern males in both treatments (Porcelli et al., 2017).

As noted above, natural selection on reproductive traits can complement or oppose sexual selection, driving these traits toward or away from their sexually selected optima. Again, research has focused on pre‐ (or peri‐) rather than postcopulatory traits. For example, cuticular hydrocarbon profiles (Sharma et al., 2012) and male genital shape (House et al., 2013) both responded differently to selection under increased temperature versus under polyandry in *D*. *simulans*, indicating divergent natural and sexual selective pressures. These forces acted convergently, however, on male genital size, which increased under both selection regimes (House et al., 2013). Male genital morphology has also been shown to be under natural selection in the cactophilic flies *D*. *buzzatii* and *D*. *koepferae* (Padró et al., 2019) and in *Gambusia* mosquitofish (Heinen‐Kay et al., 2014). Whether the same is true for sperm traits is an open question (Reinhardt et al., 2015).

The effect of natural selection on sexually selected traits has implications not only for the speed and direction of trait evolution, but also for speciation. If different environments drive divergence in trait or preference values, or if optimal values are environmentally dependent, reproductive isolation can arise between populations (Rundle & Nosil, 2005; Servedio & Boughman, 2017). This process may be accelerated if natural and sexual selection operate synergistically on a given trait (Maan & Seehausen, 2011). While the role of precopulatory barriers in preventing gene flow between environmentally diverged populations is widely recognized (Boughman, 2001; Nosil, 2012; Rundle & Nosil, 2005; Safran et al., 2013), the ability of postcopulatory traits to drive ecological speciation is less well understood (Kaufmann et al., 2015).

Here, we investigated the effect of different environments, both immediately and over the course of divergent selection, on sperm competition success. To facilitate the discovery of effects even in the case of small effect sizes, we chose two extreme conditions, rather than natural variation, as models for adaptation to novel environments: hypoxia, or low oxygen (10%, roughly half of normal levels) and hypercapnia, or high CO_2_ (5%, roughly 100 times higher than normal). Using replicate lines of *D*. *melanogaster* selected under hypoxia for more than 50 generations or under hypercapnia for more than 75 generations, we evaluated two standard measures of sperm competition: the proportion of offspring sired by the first of two males to mate with a female (P1, or sperm defense) and the proportion sired by the second male (P2, or sperm offense). We tested for local adaptation by measuring male performance under both control and selection environmental conditions.

## 2 MATERIALS AND METHODS

### 2.1 Line maintenance and treatment

All *Drosophila melanogaster* lines were derived from a wild population described in MacLellan et al. (2009). Flies were kept on a standard cornmeal–yeast–sugar medium (corn 90 g/l, agar 12 g/l, sugar 100 g/l, yeast 40 g/l, nipagin 20 ml/l, propionic acid 3 ml/l) at 25°C, 60% relative humidity, and a 12:12h light:dark cycle. Lines were maintained as non‐overlapping 14‐day generations in ten 50 ml vials per line. Every 14 days, adult flies from the ten vials were pooled and 10–15 males and 10–15 females were placed in each of ten new vials. These adults were allowed to lay eggs for 2 days before being discarded.

#### 2.1.1 Hypoxic flies

Four independent lines were generated and maintained in a hypoxic environment (approx. 10% O_2_:90% Ar) for over 50 generations, as started by and described in Charette et al. (2011). The environment was established in airtight acrylic‐plastic chambers (19 cm × 19 cm × 13 cm) with an inlet connected via plastic tubing to the gas‐mixing delivery system and an outlet allowing a constant gas flow to prevent accumulation of moisture in the plastic chambers. The oxygen/argon mixture was produced using flowmeters (Gilmont Instruments Inc, Barrington, IL, USA, and GMR Gross‐Mess‐Regeltechnik, Zella‐Mehlis, Germany) and calibrated by measuring the O_2_ concentration within the chambers using a FOXY coated fiber optic O_2_ sensor (Ocean Optics, Dunedin, FL, USA) (Charette et al., 2011).

#### 2.1.2 Hypercapnic flies

Four independent lines were generated and maintained in a hypercapnic environment (approx. 5% CO_2_ enriched ambient air) for over 75 generations. The environment was established in airtight chambers as described above. Gas mixture was regulated and measured with a gas mixer (2 CH GMix, in‐house product, University of Ottawa and GMR Gross‐Mess‐Regeltechnik, Zella‐Mehlis, Germany). Adaptation to the hypercapnic environment was tested by measuring time to incapacitation under CO_2_ anesthetization and subsequent time to recovery (see Supplementary Methods in the Appendix).

#### 2.1.3 Control flies

Eight independent lines were generated and maintained under ambient air conditions (normoxic, approx. 21% O_2_ and 0.04% CO_2_). Four lines were assigned as controls for the hypoxic lines and four as controls for the hypercapnic lines.

#### 2.1.4 Competitor males and tester females

A population of competitor flies with a recessive brown eye mutation (*bw*) was also established. Rearing conditions were the same as for the control flies except that each new generation was established with approximately 150 flies in each of two 500 ml bottles.

### 2.2 Postcopulatory reproductive success

Virgin males and females were collected on two successive days and separated by sex in 500 ml bottles (approx. 120 flies per bottle) on 100 ml of standard medium with additional live yeast as a food source. At the start of each of the two experiments (hypoxia and hypercapnia), flies were at least 3 days old. Matings were set up in 50‐ml plastic vials containing 10 ml of standard medium with live yeast.

For the sperm defense (P1) experiment, each *bw* tester female mated first with a wild‐type focal male (control or selected) and second with a *bw* competitor male. For the sperm offence (P2) experiment, each *bw* tester female mated first with a *bw* competitor male and second with a wild‐type focal male (control or selected). The female was kept together with the first male for 2 days, then transferred on day 3 to a new vial along with the second male. The female and the second male were kept together for another 2 days. On day 5, the second male was discarded and the female was transferred to a new 50 ml vial containing 10 ml of standard medium with live yeast. In the hypoxia experiment, females were again transferred to a new vial on day 7. After day 8, all females were discarded.

Mating and egg laying took place under control (normoxic) or selection (hypoxic or hypercapnic) ambient conditions. In each of the two selection experiments, males from both the control and the selected lines were tested under both control and selection ambient conditions, for a full factorial design. Females assigned to hypoxic and hypercapnic ambient conditions were exposed to normoxic ambient conditions for up to 2 h each time they were transferred to a new vial, as handling in the selection environment was not possible. After females were removed, vials were stored under standard rearing conditions and flies were allowed to develop to adulthood for subsequent scoring (see below).

From each of the 16 focal lines (i.e., four hypoxic and four hypercapnic lines, each with four control lines), 60 males were set up in each environment (hereafter ambient environment) and each role (first or second male), for a total of 3840 focal males: 1920 for the hypoxia and 1920 for the hypercapnia experiment. Each of these two experiments was split into three consecutive blocks over 6 weeks, with 20 males per line in each role and environment per block. The hypoxia experiment was conducted from February to April 2011, the hypercapnia experiment from January to March 2012. The same individuals (RD and KK) performed both experiments.

Paternity success of focal males was measured as the proportion of total offspring sired: P1 for males mated in the first (defensive) position and P2 for males mated in the second (offensive) position. To calculate P1 and P2, all offspring sired from day 3 to day 8 were scored by eye color: the focal male's offspring had wild‐type eyes, while the competitor male's offspring had brown eyes. Each vial was scored twice, on day 11 and day 13 after mating, so as to not miss any eclosed offspring. Note that it is possible that some females laid eggs on days 3–5 before mating with the second male, which would increase P1 measures (see Discussion).

Because some females may have laid eggs on days 3–4 before mating with the second male, it is possible that the analysis of all offspring from eggs laid on days 3–8 may overestimate P1 and underestimate P2. We, therefore, re‐ran the analysis to include only those offspring produced on days 5–8, after all included females had mated with both males. However, it should be noted that this approach may underestimate P1 for those females that mated immediately with the second male, if P1 decreases over time (e.g., (Chen et al., 2019); though see (Dobler & Reinhardt, 2016)).

Because offspring number may have been influenced not only by sperm competitive success but also by post‐zygotic factors, the effects of selection treatment and rearing environment on offspring survival to adulthood were also tested in a separate experiment (see Supplementary Methods in the Appendix). In addition, two responses to selection were measured: ability to withstand or recover from CO_2_ knockout; and body size (see Supplementary Methods in the Appendix). Figures were constructed using the ggplot2 (Wickham, 2016) and yarrr (Phillips, 2018) packages in R version 4.0.3 (R Core Team, 2020).

### 2.3 Statistical analysis

Data were analyzed using generalized linear mixed models (GLMMs) in the lme4 package (Bates et al., 2015) in R version 4.0.3 (R Core Team, 2020). Models had a binomial distribution with logit‐link error function; the dependent variable, offspring proportion, was made independent of sample size by using the *cbind* function. The predictors were selection treatment, mating environment, and their interaction, with line and experimental block as random factors. Mating pair ID was also included as a random factor to correct for overdispersion (Harrison, 2014). Sum‐to‐zero contrasts were used for the fixed effects (Levy, 2014).

## 3 RESULTS

In the hypoxia experiment, 1413 out of the 1920 females produced offspring. Of these, 1185 females had offspring sired both by the first and by the second male to mate. The remaining cases, where P1 = 1 and P2 = 0 (*n* = 195) or vice versa (*n* = 33), were excluded, since some of these males may not have mated or successfully transferred sperm. There was no difference in the likelihood of control versus hypoxia males to sire any offspring when they were the first male to mate (Χ^2^ = 0.53, *p* = .47), but hypoxia males were less likely to do so than control males when in the second mating role (Χ^2^ = 20.02, *p* < .0001). In the hypercapnia experiment, 1677 out of the 1920 females produced offspring, with 1407 having offspring from both males (P1 = 1, P2 = 0: *n* = 191; P1 = 0, P2 = 1: *n* = 79). Control males were less likely than hypoxia males to sire any offspring in the first role (Χ^2^ = 7.61, *p* = .006), but there was no difference in the second role (Χ^2^ = 0.04, *p* = .83).

**FIGURE 1:**
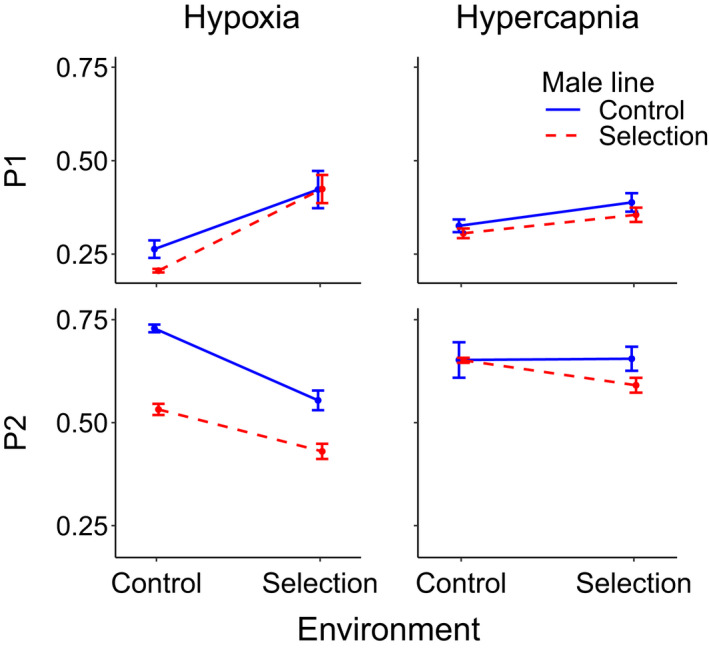
Proportion of offspring sired by the first (P1, sperm defensive ability) and second (P2, sperm offensive ability) male to mate with a female. Hypoxia‐selected and corresponding control males competed against non‐focal *bw* males under both hypoxic and control ambient environmental conditions; hypercapnia‐selected and corresponding control males did so under both hypercapnic and control ambient environmental conditions. Bars show standard errors across the four replicate means

Summary statistics for P1 and P2 are shown in Table 1; GLMM results are shown in Table 2 and Figure 1, with individual results for each line shown in Figures [Link], [Link], [Link], [Link]. In the hypoxia experiment, selection under hypoxia decreased both P1 and P2 (Figures A1 and A2), while a hypoxic ambient environment increased P1 and decreased P2 (Figures A3 and A4). For P1, there was also a significant interaction between selection treatment and environment, such that hypoxia‐selected males sired fewer offspring than control males in the control environment but not in the hypoxic environment. In the hypercapnia experiment, selection treatment had no effect on either P1 or P2. A hypercapnic ambient environment had a positive effect on P1 but no effect on P2. For P2, there was a trend (*p* < .10) for an interaction between selection treatment and environment, such that hypercapnia‐selected males did worse under hypercapnic than under normoxic conditions, while control males were unaffected by ambient environment.

**TABLE 1:** Summary statistics (mean ± *SD* [*n*]) for P1 and P2 in the hypoxia and hypercapnia experiments

Male line	Ambient environment	P1	P2
Control	Control	0.263 ± 0.179 [200]	0.729 ± 0.182 [190]
Control	Hypoxia	0.423 ± 0.260 [110]	0.554 ± 0.266 [118]
Hypoxia	Control	0.206 ± 0.172 [207]	0.532 ± 0.268 [173]
Hypoxia	Hypoxia	0.424 ± 0.255 [98]	0.430 ± 0.245 [89]
Control	Control	0.326 ± 0.212 [187]	0.652 ± 0.217 [172]
Control	Hypercapnia	0.388 ± 0.221 [172]	0.655 ± 0.198 [173]
Hypercapnia	Control	0.306 ± 0.176 [190]	0.651 ± 0.172 [168]
Hypercapnia	Hypercapnia	0.355 ± 0.200 [172]	0.591 ± 0.197 [173]

**TABLE 2:** GLMM results for effects of selection treatment and ambient environment on P1 and P2 in the hypoxia and hypercapnia experiments. Significant predictors are shown in bold

	Est	*SE*	*t*‐Value	*p*‐value
Hypoxia experiment				
P1				
Intercept	−1.424	0.08	−17.78	<1e−15
**Selection treatment (hypoxia)**	−0.210	0.088	−2.392	.017
**Ambient environment (hypoxia)**	0.569	0.067	8.535	<.0001
**Treatment x environment**	0.298	0.133	2.244	.025
P2				
Intercept	−0.726	0.063	−11.60	<1e−15
**Selection treatment (hypoxia)**	−0.360	0.058	−6.227	<.0001
**Ambient environment (hypoxia)**	−0.251	0.058	−4.318	<.0001
Treatment × environment	0.190	0.117	1.626	.104
Hypercapnia experiment				
P1				
Intercept	−1.235	0.039	−31.734	<1e−15
Selection treatment (hypercapnia)	−0.044	0.078	−0.560	.575
**Ambient environment (hypercapnia)**	0.177	0.043	4.117	<.0001
Treatment × environment	−0.079	0.086	−0.912	.362
P2				
Intercept	−0.514	0.033	−15.735	<1e−15
Selection treatment (hypercapnia)	−0.031	0.065	−0.482	.630
Ambient environment (hypercapnia)	−0.043	0.034	−1.27	.204
Treatment × environment	−0.128	0.068	−1.868	.062

Results of the analysis including only those offspring from eggs laid on days 5–8 are shown in the Appendix. As expected, P1 decreased and P2 increased (Table A1) compared to the analysis of offspring from eggs laid on days 3–8. The direction of nearly all model coefficients remained the same, though some effects lost significance and others attained it (Table A2).

Neither selection treatment nor its interaction with rearing environment had an effect on the egg‐to‐adult ratio, suggesting that differential offspring survival did not bias paternity estimates (Table A3; although there was a trend for a negative effect of ambient hypoxia, such an effect would not introduce a bias). There was no main effect of selection treatment on adult offspring number (Table A4) or on ability to withstand or recover from CO_2_ knockout (Table A3, Figures A5–A8). Selection under both hypoxia and hypercapnia decreased body size in both sexes (Table A6, Figure A9).

## 4 DISCUSSION

Sperm competitive ability, like other sexually selected traits, may also be subject to natural selection, potentially leading to local ecological adaptation and population divergence. Here, we found that sperm competition success, both defensive and offensive, changed with selection under hypoxia but not under hypercapnia. However, evidence for local adaptation was limited: while the positive effect of an ambient hypoxic environment on P1 was greater in hypoxia‐selected than in control males, the P1 value itself under hypoxia was no greater in selected than in control males. Rather, selection under hypoxia led to a general decrease in sperm competitiveness across most mating (first vs. second male) and environmental (hypoxia vs. control) contexts.

### 4.1 Little evidence of adaptation in selected lines

Evidence for adaptation to hypoxia was likewise mixed in a previous study using the same populations tested here (Charette et al., 2011). Hypoxia‐selected flies showed acute hypoxia tolerance, taking longer than control flies to become incapacitated by argon gas. They also evolved increased activity of citrate synthase, the enzyme responsible for initiating the citric acid cycle. This finding may be indicative of increased mitochondrial density, though the resting metabolic rate was unchanged. However, in the Charette et al. study, a hypoxic ambient environment decreased survival and offspring production in hypoxia‐selected flies as much as it did in control flies.

In hypercapnia‐selected flies, we again found limited evidence for adaptation. Compared to controls, these flies had longer times to incapacitation under CO_2_ anesthetization (females) and shorter times to subsequent recovery (males), consistent with adaptation. However, flies selected under hypoxic conditions also had longer times to incapacitation (males) and shorter recovery times (females) than did controls, despite no history of selection under hypercapnia (see Supplementary Results in the Appendix). Furthermore, a hypercapnic ambient environment tended to negatively impact P2 in hypercapnia‐selected but not control males, indicating maladaptation to the selection environment. While it is certainly possible that we would have detected adaptation in the hypoxia‐ and/or hypercapnia‐selected lines had we measured a different fitness component, it may also be that our harsh selection environment imposed high fitness costs that outweighed any fitness benefits. In support of this idea, we previously showed that the hypercapnic lines had decreased egg and offspring production, regardless of whether the ambient environment was hypercapnic or normoxic (K. Reinhardt, D. Cassens, B. Turnell, R. Dobler, unpublished data).

### 4.2 Decreased postcopulatory success in hypoxia‐selected flies

We observed lower sperm competitiveness in hypoxia‐selected flies compared to controls. Such declines in reproductive fitness during artificial selection are common and have long been recognized in animal breeding (Latter & Robertson, 1962). In *D*. *melanogaster*, for example, selection under nutritional stress decreased male mating success, while in the Mexican fruit fly *Anastrepha ludens*, selection for desiccation resistance led to smaller accessory glands and seminal vesicles and to decreased female storage of sperm from these males (Pérez‐Staples et al., 2018).

Several factors may explain the decreased sperm competitiveness of hypoxia‐selected males in our study. First, 33 genes in *D*. *melanogaster* have been directly implicated in sperm competitiveness (reviewed in Civetta & Ranz, 2019), and some of these genes, or genes with which they were linked, may have been targeted during selection. Second, body size declined in response to selection under hypoxia both in our experiment and in two other studies on *D*. *melanogaster* (Henry & Harrison, 2004; Zhou et al., 2007). While selection under hypercapnia also led to decreased body size, the effect was only half as strong. Smaller males have been shown to have lower sperm competition success in this species, in both the defensive (McGraw et al., 2007) and the offensive (Bangham et al., 2002; though see Travers et al., 2016) roles. This pattern may be due to small males having shorter sperm (Amitin & Pitnick, 2007; though see Lüpold et al., 2016), which are less able both to displace a competitor's sperm and to resist displacement (Lüpold et al., 2012; Miller & Pitnick, 2002; Pattarini et al., 2006).

A third possible explanation for the reduced sperm competitiveness in hypoxia‐selected flies is a—possibly transient—decline in male condition during selection. Evidence for condition dependence of sperm traits in *D*. *melanogaster* is mixed: while male nutrition during the larval (Morimoto & Wigby, 2016) or adult (Fricke et al., 2008) stages does not affect P1 or P2, increased larval rearing density decreases P1 (Amitin & Pitnick, 2007). In addition, sperm production is condition dependent across *Drosophila* species (Lüpold et al., 2016). If male body size and/or condition declined during selection, the lower postcopulatory success we observed in hypoxia‐selected males could be due either to their ejaculates being intrinsically less competitive, for the reasons outlined above, or to cryptic female choice for larger or higher condition males (Firman et al., 2017). *Drosophila* females can bias paternity toward preferred mating partners through several mechanisms, including sperm ejection, differential sperm storage, and the timing of remating and of oviposition (Ala‐Honkola & Manier, 2016; Manier, Lüpold, Belote, et al., 2013; Manier et al., 2013); and even, potentially, the differential processing of seminal fluid proteins (Sirot & Wolfner, 2015). Such post‐mating biases may account for the high P2 levels achieved by attractive *D*. *simulans* males (Hosken et al., 2008), and for the correlations between both P1 and P2 and mating success in *D*. *melanogaster* (Fricke et al., 2010; though see Pischedda & Rice, 2012).

Fourth, the decreased sperm competitiveness of the hypoxia‐selected flies could have been caused by drift, if selection decreased the effective population size (Frankham et al., 1988). In our experiment, selection under hypoxia may have decreased not only the effective population size but also the census size: although the effect was marginally non‐significant, adult offspring production was lower in hypoxia‐selected compared to control lines (see Table A4).

Fifth, the hypoxia‐selected males’ decreased sperm competitiveness was most evident in their lower P2 values. As noted in the results, these males were less likely than control males to sire any offspring at all in the second mating role, suggesting that some of these males may not have mated at all. It is also possible that those hypoxia‐selected second males that did sire offspring took longer, on average, than control males to mate with the female. In this case, the lower P2 of hypoxia‐selected males may be due, in part, to increased mating latency, which would give the first males’ sperm more time to fertilize the females’ eggs in the absence of sperm competition.

Finally, as hypoxia‐selected flies were reared in a hypoxic environment, it is possible that immediate environmental effects contributed to their decreased sperm competitiveness and/or decreased body size.

### 4.3 A potential role for reactive oxygen species

The low postcopulatory success of hypoxia‐selected males may also be due to their sperm potentially being subject to greater oxidative stress from reactive oxygen species (ROS) than the sperm of control males. ROS are formed when electrons flowing down the mitochondrial electron transport chain are transferred to molecular oxygen instead of to the next subunit (Balaban et al., 2005). If not neutralized by antioxidants, they can react with and cause damage to cells; and sperm are particularly susceptible due to their limited antioxidant reserves and to the high concentration of oxidation‐prone polyunsaturated fatty acids in their membranes (Aitken, 2020).

*Drosophila melanogaster* selected under hypoxia have been shown to increase their use of Complex I relative to Complex II of the electron transport chain during oxidative phosphorylation (Feala et al., 2009; Zhou et al., 2007). While increasing the amount of ATP produced per unit oxygen consumed, this shift may also increase oxidative stress, as Complex I is a major site of mitochondrial ROS production in this species (Miwa et al., 2003). *D*. *melanogaster* sperm use oxidative phosphorylation for energy metabolism (Turnell & Reinhardt, 2020), and they produce mitochondrial ROS (Turnell & Reinhardt, 2020) at environment‐dependent rates (Guo & Reinhardt, 2020). If the flies in our study responded to selection under low oxygen by boosting their respiratory efficiency through increased use of Complex I, they likely experienced elevated ROS levels, including in their sperm.

High ROS levels have been shown to impair sperm function, including motility, viability, and fertilization capacity, across taxa (Baumber et al., 2000; Hagedorn et al., 2012; Garratt et al., 2013; Reinhardt & Ribou, 2013; Morielli & O’Flaherty, 2015; see also Friesen et al., 2020). ROS can also damage sperm DNA, leading to embryo inviability (Lane et al., 2014; Tremellen, 2008), and thus decreased paternity success, although this pattern is unlikely to explain our observations since selection treatment did not affect egg‐to‐adult survival. Proximal hypoxia also increases oxidative stress, both in sperm cells (Castro et al., 2020) and in the somatic tissues of the male reproductive system (Torres et al., 2014), thereby impairing spermatogenesis (Farias et al., 2010).

### 4.4 Contrasting effects of ambient environment on P1 and P2

P1 increased under both artificial ambient environments. In contrast, P2 decreased under hypoxia (control and selected males) and under hypercapnia (selected males only). We can think of three possible explanations for this result. First, rates of aerobic metabolism and ROS production in sperm may have been lower under hypoxic and hypercapnic conditions. If so, the sperm of the first male to mate would have accrued less oxidative damage in female storage under artificial than under control conditions, and would, therefore, have been better able to compete with the fresh, incoming sperm of the second male. In support of this idea, honeybee (*Apis mellifera*) sperm have been shown to switch from oxidative phosphorylation to the less‐damaging glycolysis in the anaerobic environment of the female spermatheca (Paynter et al., 2017). Prolonged exposure to hypoxia also caused a glycolytic shift in the testes of medaka fish (Wang et al., 2016). On the other hand, oxygen consumption increased rather than decreased in the sperm of rats exposed to chronic hypoxia (Farias et al., 2005) and in fish sperm under hypoxic conditions (Castro et al., 2020; Fitzpatrick et al., 2009).

Second, sperm velocity may have declined under ambient hypoxia and hypercapnia. In *D*. *melanogaster*, slower sperm, like longer sperm, are better able to displace rival sperm and to resist being displaced (Lüpold et al., 2012). If the negative effect of a hypoxic or hypercapnic storage environment on sperm velocity is cumulative, or takes some time to occur, then this effect would have been greater in the first male's sperm. Decreased oxygen has been shown to reduce sperm swimming speed in a variety of taxa (Graham et al., 2016; He et al., 2015; Shin et al., 2014), although the effect of elevated CO_2_ can be negative (Munday et al., 2019) or positive (Graham et al., 2016; Wandernoth et al., 2010).

Third, ambient hypoxia and hypercapnia during mating and egg laying may have altered female reproductive physiology or behavior in such a way as to favor the first male over the second. For example, hypoxia and hypercapnia may have exerted a cumulative or time‐dependent effect on females such that sperm uptake or storage was progressively reduced or sperm ejection was progressively increased.

Interestingly, the increased P1 and decreased P2 (in selected males) under hypercapnia we found here contrasts directly with our finding in a previous study, in which P1 decreased and P2 increased under elevated CO_2_ (Dobler & Reinhardt, 2016). As the gas conditions and source population in that experiment were the same as used here, the reasons for this difference remain unclear. However, substantial differences in patterns of competitive fertilization success have been reported before, even across replicates within a single experiment (e.g., Amitin & Pitnick, 2007). Thus, while sperm precedence patterns in the current study varied minimally across the four lines within each of the four selection treatments (i.e., hypoxia, hypercapnia, and their respective controls; Figures [Link], [Link], [Link], [Link]), the variation observed across experiments is perhaps not surprising.

## 5 CONCLUSION

In summary, we found evidence that the environment shapes sperm competitive success, a sexually selected fitness trait, but little evidence of local adaptation. Environment affected the defensive and offensive abilities of sperm in general, and in largely similar ways for selected and control males’ sperm. It remains to be determined what specific sperm and ejaculate phenotypes (such as sperm number, morphology, viability, and motility; and seminal fluid protein quantity and quality (Ramm, 2020)) underlie these changes in sperm competitiveness, and whether and how they trade off with one another during the course of selection (e.g., Cardozo et al., 2020).

Our study has several limitations. It seems likely that our harsh selection environments imposed costs that masked any adaptation. In addition, because the experimental generation and the immediately previous generation were reared in their respective selection environments rather than in a common‐garden environment, we cannot rule out the effects of plasticity on the phenotypic differences we found.

Future work is needed to determine the extent to which natural selection can shape postcopulatory traits and drive population divergence through postcopulatory mechanisms. Given the important role played by cryptic female choice in many of these mechanisms, investigating the effect of natural selection on female sperm use patterns, as well as on sperm traits, would be informative.

## CONFLICT OF INTEREST

The authors declare no conflict of interest.

## AUTHOR CONTRIBUTION

**Ralph Dobler:** Conceptualization (equal); Data curation (lead); Formal analysis (lead); Funding acquisition (equal); Investigation (equal); Methodology (equal); Project administration (equal); Supervision (equal); Visualization (equal); Writing – original draft (equal); Writing – review & editing (supporting). **Marc Charette:** Resources (equal). **Katrin Kaplan:** Investigation (equal). **Biz R. Turnell:** Formal analysis (supporting); Visualization (equal); Writing – original draft (equal); Writing – review & editing (lead). **Klaus Reinhardt:** Conceptualization (equal); Funding acquisition (equal); Methodology (equal); Project administration (equal); Resources (equal); Supervision (equal); Writing – review & editing (supporting).

## AUTHORS’ CONTRIBUTIONS

RD and KR conceived the study. MC established the control and selection lines. RD and KK performed the experiments and RD and BRT analyzed the data. RD and BRT drafted the manuscript, with revisions by KR; all authors approved the final version.

## Supporting information

Supplementary Material

Supplementary Material

Supplementary Material

Supplementary Material

Supplementary Material

Supplementary Material

Supplementary Material

Supplementary Material

Supplementary Material

Appendix S1

## Data Availability

Data are archived in the Dryad Digital Repository (https://doi.org/10.5061/dryad.j9kd51cf2).
