# Supplementary figures and images for: Divergent natural selection alters male sperm competition success in Drosophila melanogaster
Source: Ecol Evol. 2022 Feb 16;12(2):e8567. doi: 10.1002/ece3.8567 (PMC8848461; doi:10.1002/ece3.8567)

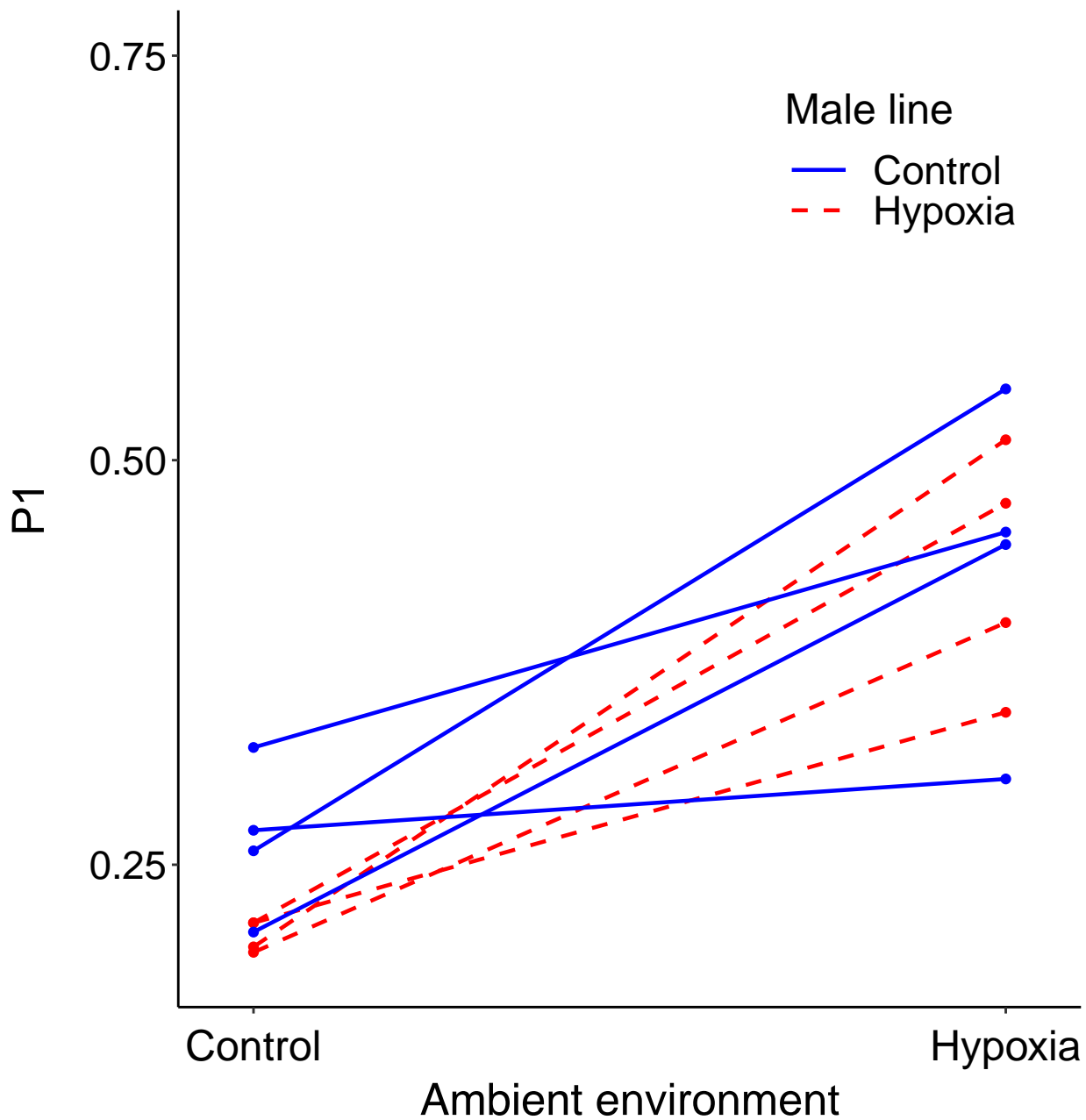

Supplement: Supplementary file 1 — Supplementary Material [file ECE3-12-e8567-s002.pdf]

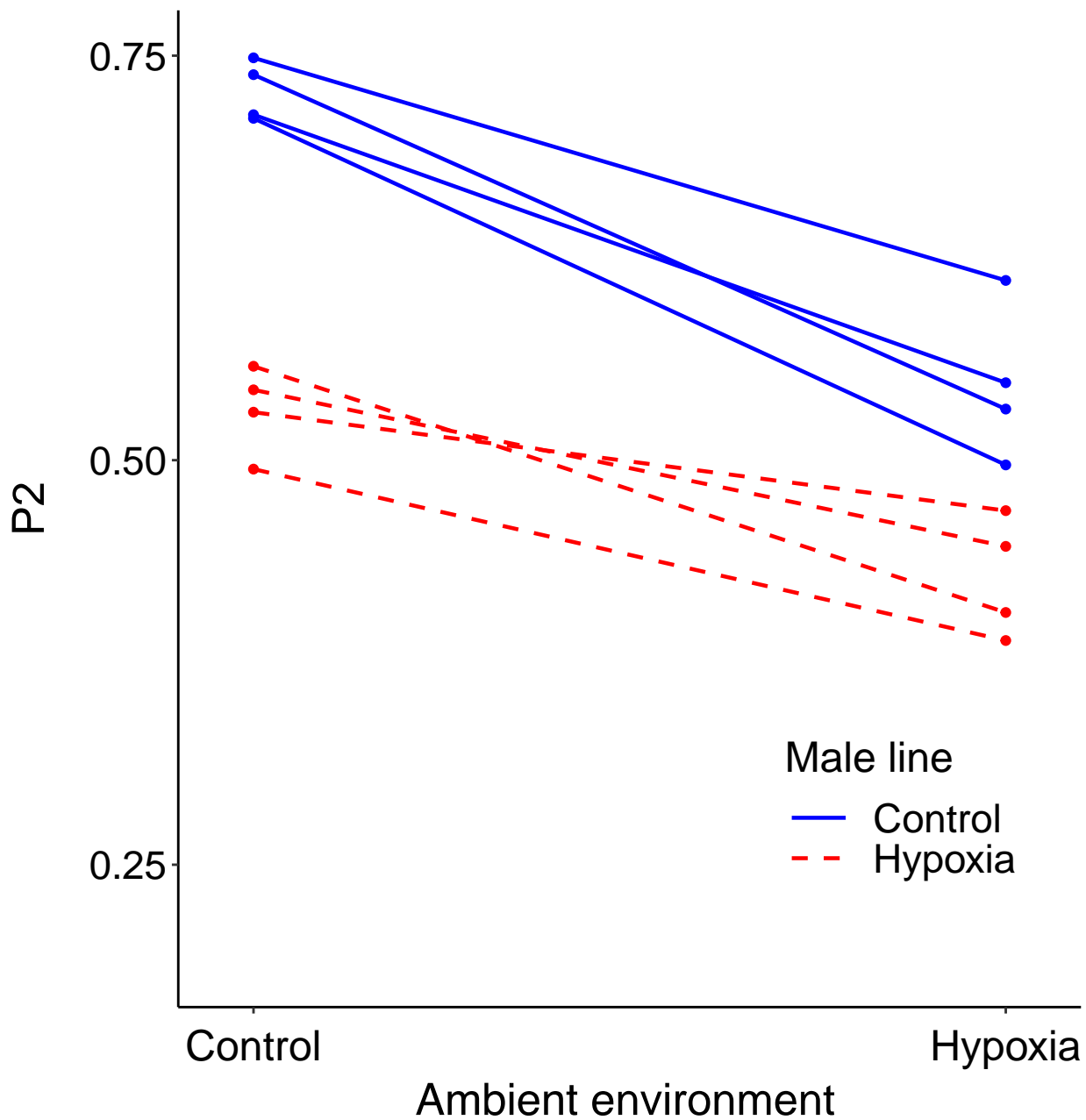

Supplement: Supplementary file 2 — Supplementary Material [file ECE3-12-e8567-s009.pdf]

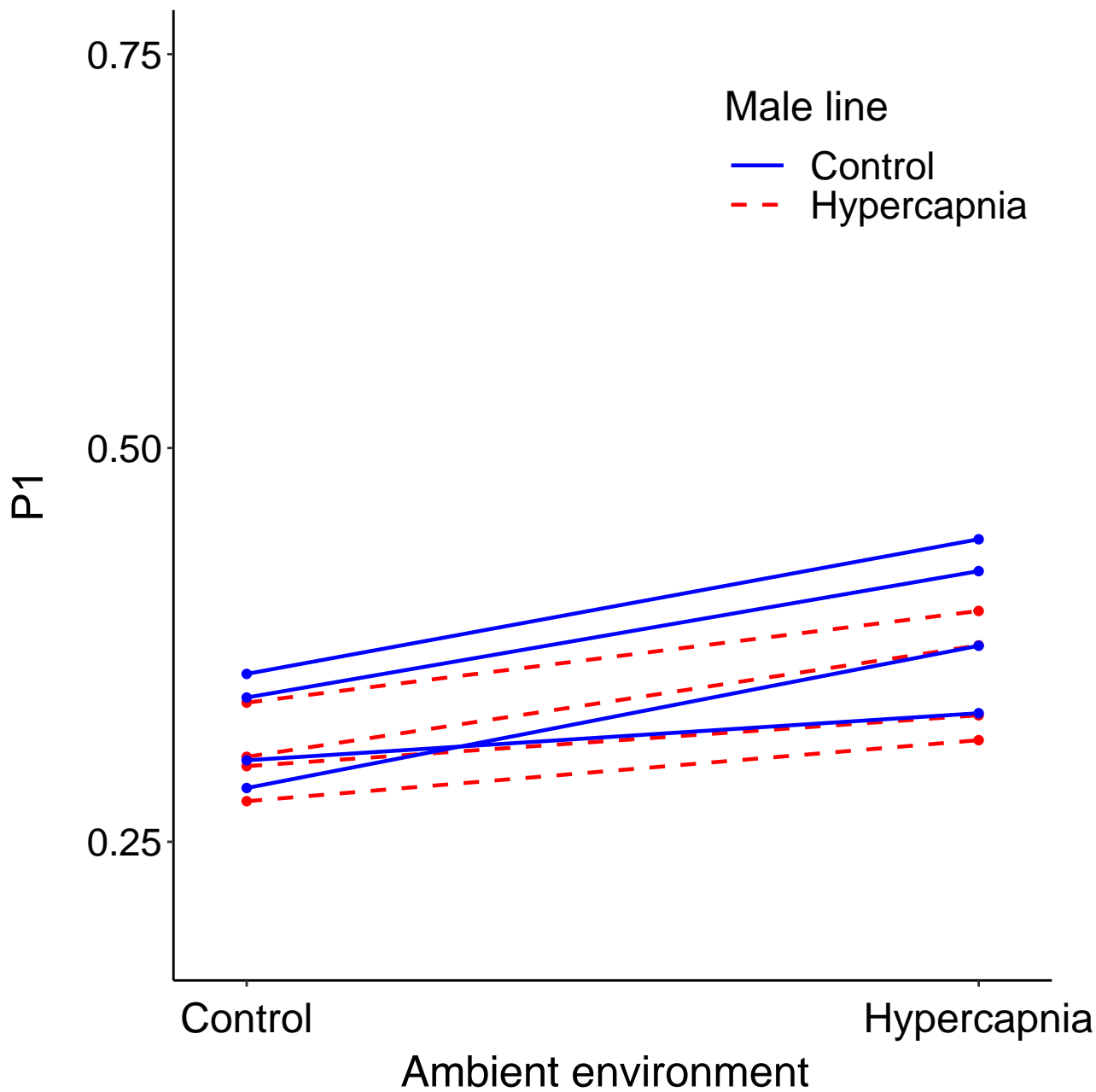

Supplement: Supplementary file 3 — Supplementary Material [file ECE3-12-e8567-s006.pdf]

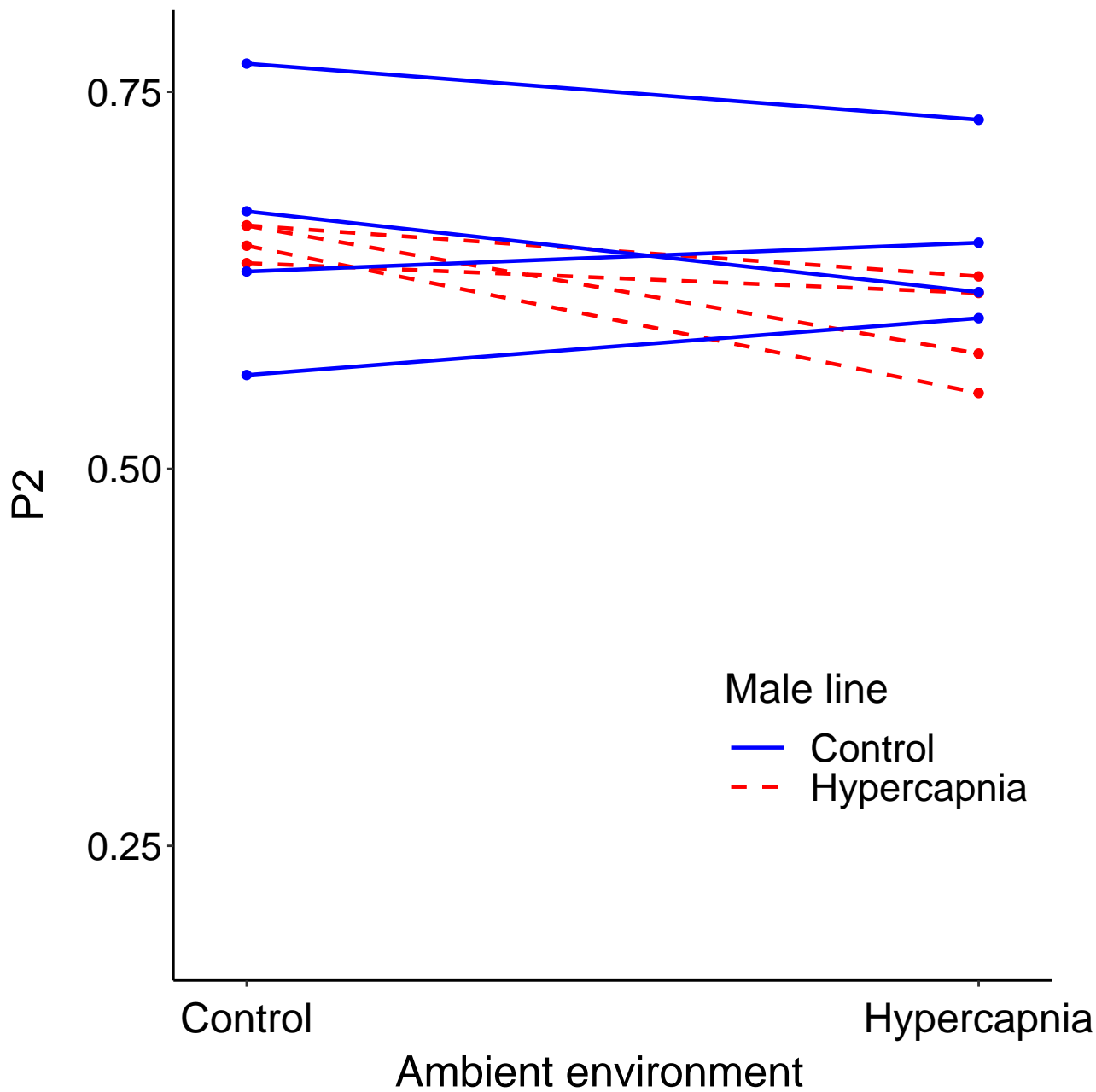

Supplement: Supplementary file 4 — Supplementary Material [file ECE3-12-e8567-s003.pdf]

Experiment = Hypercapnia

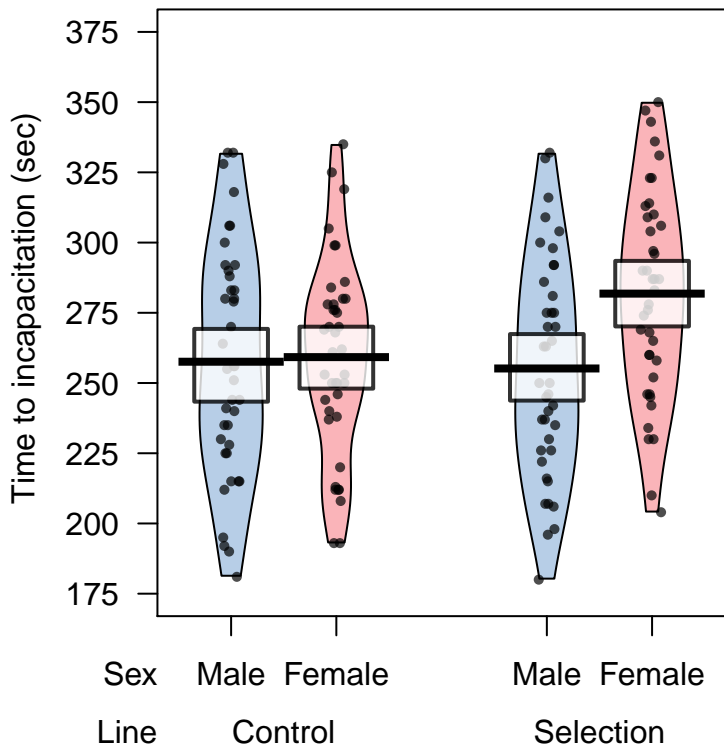

Experiment = Hypoxia

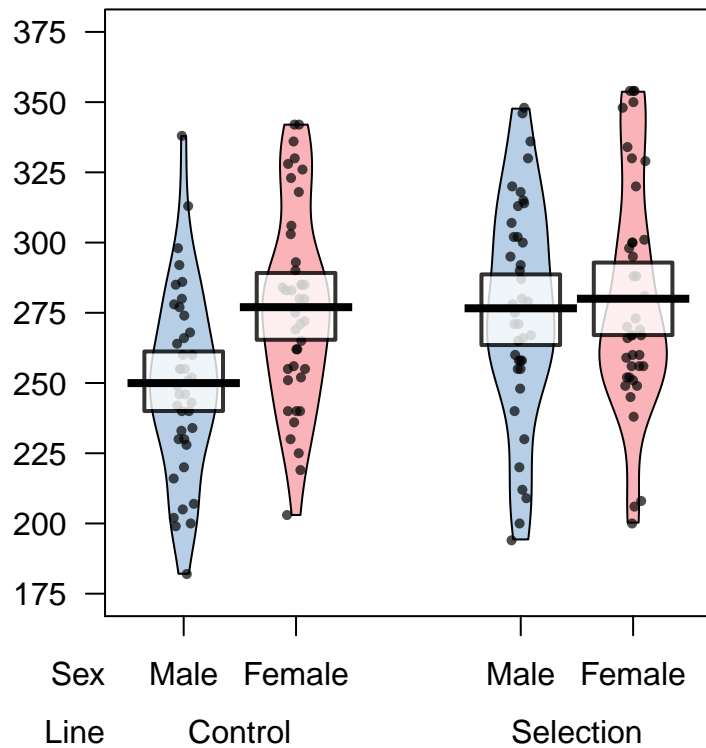

Supplement: Supplementary file 5 — Supplementary Material [file ECE3-12-e8567-s007.pdf]

Experiment = Hypercapnia

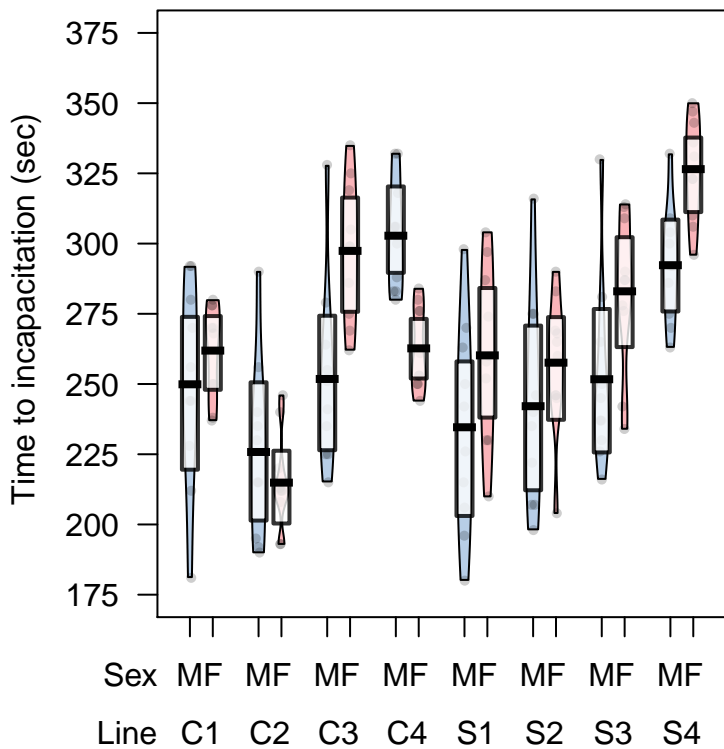

Experiment = Hypoxia

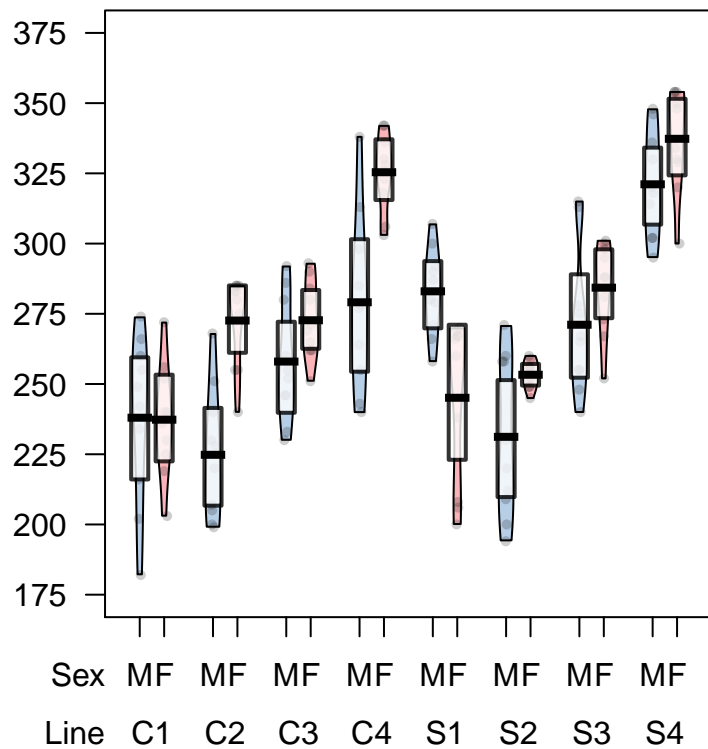

Supplement: Supplementary file 6 — Supplementary Material [file ECE3-12-e8567-s008.pdf]

Experiment = Hypercapnia

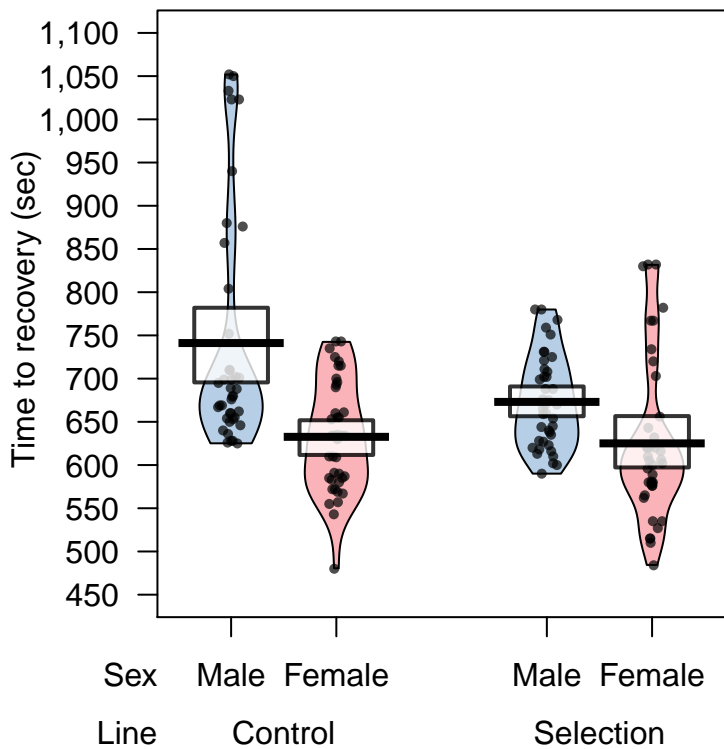

Experiment = Hypoxia

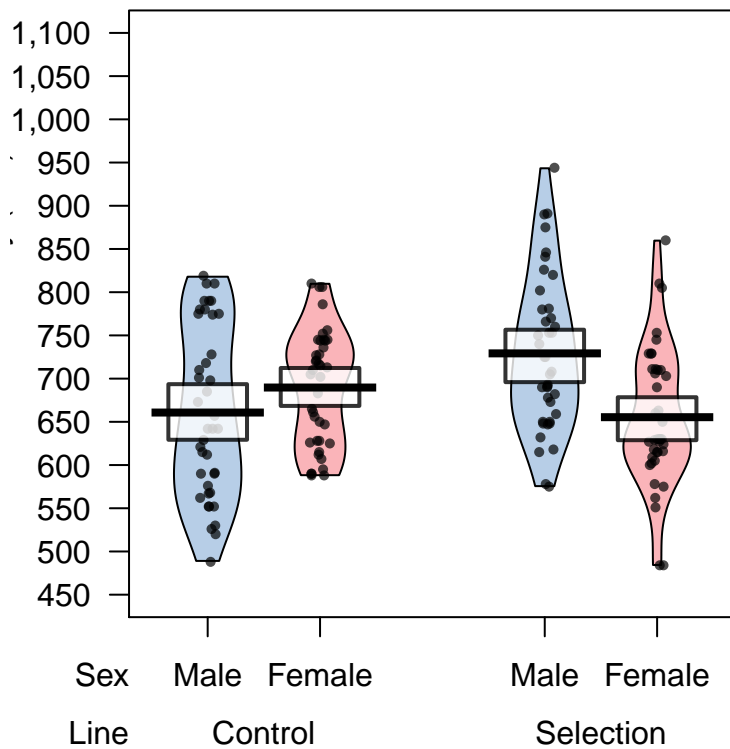

Supplement: Supplementary file 7 — Supplementary Material [file ECE3-12-e8567-s001.pdf]

Experiment = Hypercapnia

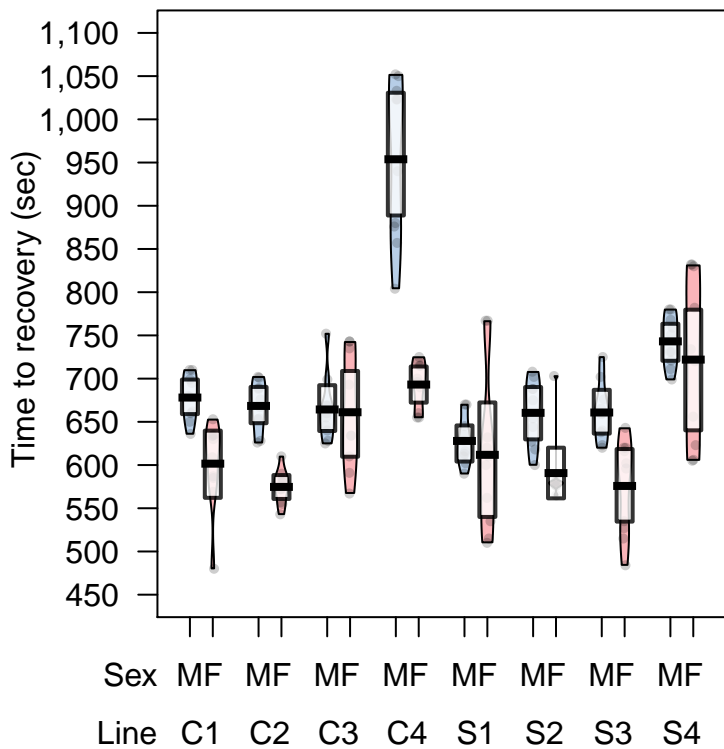

Experiment = Hypoxia

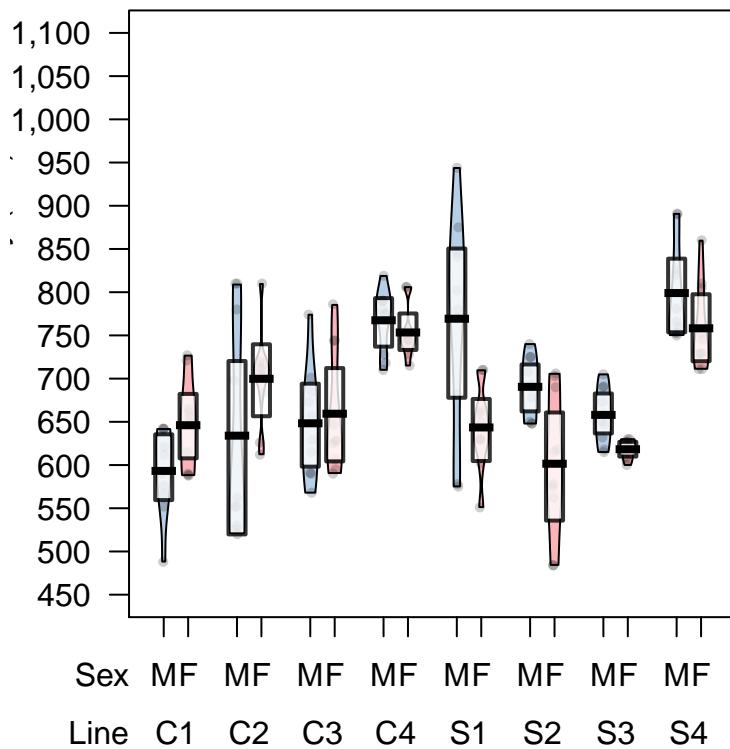

Supplement: Supplementary file 8 — Supplementary Material [file ECE3-12-e8567-s004.pdf]

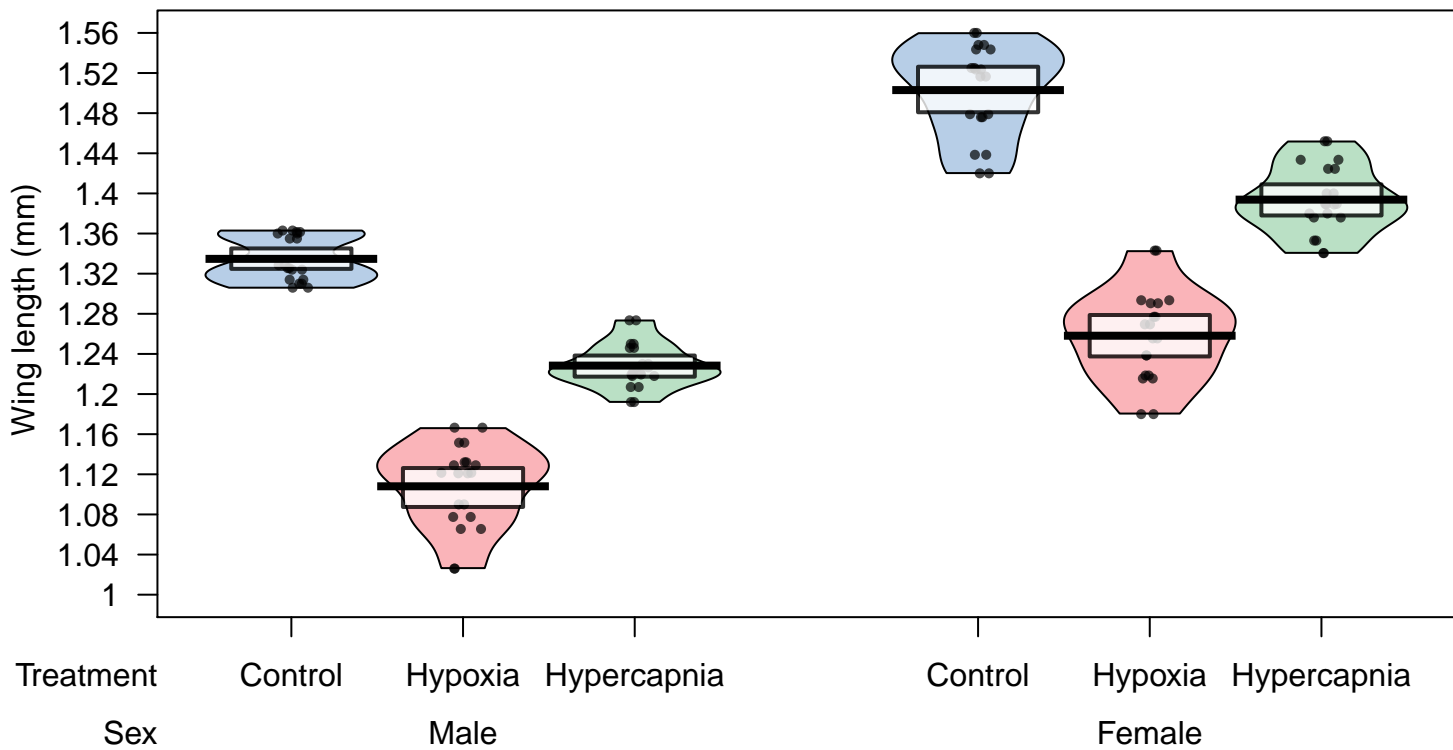

Supplement: Supplementary file 9 — Supplementary Material [file ECE3-12-e8567-s005.pdf]
