# Supplementary material for: Divergent natural selection alters male sperm competition success in Drosophila melanogaster
Source: Ecol Evol. 2022 Feb 16;12(2):e8567. doi: 10.1002/ece3.8567 (PMC8848461; doi:10.1002/ece3.8567)
Supplement: Supplementary file 10 — Appendix S1 [file ECE3-12-e8567-s010.docx]

**Appendix**

**Supplementary Methods**

*Egg-to-adult ratio and adult offspring number*

To test whether selection treatment or rearing environment has an effect on offspring development from egg to adult, 10 male/female pairs from each of the 16 lines were kept together under control conditions or selection conditions (hypoxic or hypercapnic) in a full factorial design (10 pairs x 8 lines x 2 environments = 160 pairs for each of the two experiments). On day 1 the virgin female and the virgin male were put together in a 50ml vial on 10ml of standard medium with dry yeast. On day 2 the female was transferred to a new vial, the male was discarded, and the eggs in the first vial were counted. On day 3 the female was also discarded and the eggs in the second vial were counted. On day 11 (12) and 14 (15) emerged flies per vial were counted. The ratio of emerged flies to laid eggs per female was analysed for each of the two experiments using generalized linear mixed models with binomial distributions and logit-link error functions. The dependent variable, egg-to-adult ratio, was made independent of sample size by using the *cbind* function. The predictors were selection treatment, rearing environment, and their interaction, with line as a random factor.

To test whether selection treatment may have had an effect on population size, total adult offspring number was compared between the hypoxia- or hypercapnia-selected pairs and their respective controls. Only those pairs kept in their respective treatment environment (hypoxia, hypercapnia, and control) were used (10 pairs x 8 lines = 80 pairs for each of the two experiments). Total offspring number was analysed for each of the two experiments using linear mixed models, with selection condition (control vs. selection treatment and ambient conditions) as a fixed factor and line as a random factor.

*Response to selection/adaptation*

To test whether the four hypercapnic lines showed an adaptive response to selection, flies were knocked out with CO_2_ and the following two parameters were measured: (1) time to incapacitation and (2) time to recovery, defined as standing up and walking around in the vial in response to gentle tremors (approx. every 5 seconds). The four hypoxic lines, as well as all eight control lines, were also measured. All flies had been reared and maintained in their respective treatment environments (hypoxia, hypercapnia, and control). Ten flies of each sex and line were anaesthetized individually in 50ml vials closed with foam plugs. Data were analysed separately for the hypercapnia and hypoxia experiments using two-way ANOVAs. Selection treatment (control vs. selection), sex, and their interaction were included as fixed effects and line ID as a random effect.

To test whether selection affected body size, wing length was measured in ten males and ten females from one randomly selected line in each of the three treatments (hypoxia, hypercapnia, and control, all reared and maintained in their respective treatment environments). Left and right wing lengths, as measured from the anterior crossvein to distal tip of vein III (Starmer & Wolf, 1989), were averaged for each individual. Males and females were separately compared across groups using ANOVAs with post-hoc two-way comparisons via Tukey’s HSD.

**Supplementary Results**

*Paternity share, offspring from eggs laid on days 5–8 only*

Compared to offspring from eggs produced on days 3–8, offspring from eggs produced on days 5–8 were less likely to be sired by the first male (Table A1 vs. Table 1). The direction of all model coefficients remained the same (Table A2 vs Table 2) with one exception: treatment x environment for P2 in the hypercapnia experiment, non-significant in both analyses, switched from negative to positive.

The negative effect of selection under hypoxia remained significant for P2 but not P1, while a negative effect of selection under hypercapnia on P1 emerged. The positive effects of both ambient hypoxia and ambient hypercapnia on P1 remained significant, but the negative effect of ambient hypoxia on P2 did not. Finally, the interaction between treatment and environment on P1 in the hypoxia experiment remained significant (i.e., the positive effect of ambient hypoxia was stronger for hypoxia-selected males), while the non-significant interaction between treatment and environment on P2 in the hypercapnia experiment achieved significance (i.e., the negative effect of ambient hypercapnia was stronger for hypercapnia-selected males).

**Table A1.** Summary statistics (mean ± sd [n]) for P1 and P2 in the hypoxia and hypercapnia experiments, days 5–8).

| **Male line** | **Ambient environment** | **P1** | **P2** |
| --- | --- | --- | --- |
| Control | Control | 0.060 ± 0.158 [197] | 0.926 ± 0.186 [188] |
| Control | Hypoxia | 0.056 ± 0.177 [89] | 0.875 ± 0.238 [91] |
| Hypoxia | Control | 0.040 ± 0.134 [203] | 0.719 ± 0.295 [170] |
| Hypoxia | Hypoxia | 0.090 ± 0.241 [77] | 0.739 ± 0.270 [67] |
| **Male line** | **Ambient environment** | **P1** | **P2** |
| Control | Control | 0.108 ± 0.207 [182] | 0.876 ± 0.239 [169] |
| Control | Hypercapnia | 0.149 ± 0.231 [167] | 0.895 ± 0.191 [168] |
| Hypercapnia | Control | 0.080 ± 0.189 [187] | 0.888 ± 0.179 [163] |
| Hypercapnia | Hypercapnia | 0.100 ± 0.187 [165] | 0.832 ± 0.221 [170] |

**Table A2.** GLMM results for effects of selection treatment and ambient environment on P1 and P2 in the hypoxia and hypercapnia experiments, days 5–8. Significant predictors are shown in bold. Italics indicate qualitative (i.e., significant vs. non-significant) differences compared to the results of the full analysis (Table 2).

| **Hypoxia experiment** | | **Est** | **SE** | ***t*-value** | ***p*-value** |
| --- | --- | --- | --- | --- | --- |
| **P1** | Intercept | -5.818 | 0.229 | -25.427 | < 1e-15 |
|  | *Selection treatment (hypoxia)* | -0.447 | 0.336 | -1.33 | 0.183 |
|  | **Ambient environment (hypoxia)** | 0.891 | 0.335 | 2.661 | 0.008 |
|  | **Treatment x environment** | 1.531 | 0.672 | 2.279 | 0.023 |
| **P2** | Intercept | -0.277 | 0.057 | -4.862 | < 1e-5 |
|  | **Selection treatment (hypoxia)** | -0.248 | 0.053 | -4.676 | < 1e-5 |
|  | *Ambient environment (hypoxia)* | -0.011 | 0.053 | -0.207 | 0.836 |
|  | Treatment x environment | 0.128 | 0.107 | 1.200 | 0.230 |
| **Hypercapnia experiment** | | **Est** | **SE** | ***t*-value** | ***p*-value** |
| **P1** | Intercept | -4.034 | 0.101 | -39.839 | < 1e-15 |
|  | ***Selection treatment (hypercapnia)*** | -0.383 | 0.186 | -2.056 | 0.040 |
|  | **Ambient environment (hypercapnia)** | 0.76 | 0.187 | 4.065 | < 1e-4 |
|  | Treatment x environment | 0.039 | 0.373 | 0.105 | 0.916 |
| **P2** | Intercept | -0.165 | 0.024 | -6.986 | < 1e-11 |
|  | Selection treatment (hypercapnia) | -0.02 | 0.047 | -0.427 | 0.670 |
|  | Ambient environment (hypercapnia) | -0.004 | 0.026 | -0.143 | 0.886 |
|  | ***Treatment x environment*** | -0.118 | 0.053 | -2.246 | 0.025 |

*Egg-to-adult ratio and offspring number*

There was no significant effect of selection treatment, ambient conditions, or their interaction on the egg-to-adult ratio for either the hypoxia (n = 148 pairs) or the hypercapnia (n = 148 pairs) experiment (Table A3).

There was no significant effect of treatment (control vs. extreme selection environment and ambient conditions) on total adult offspring number for either the hypoxia (n = 71 pairs) or the hypercapnia (n = 71 pairs) experiment, although there was a trend for decreased offspring production in the hypoxia lines (Table A4). Offspring number was 18% and 12% lower in the in the hypoxia and hypercapnia lines, respectively, compared to control lines (mean ± SD: 59.2 ± 18.1 vs. 72.3 ± 24.8; 69.2 ± 16.8 vs. 78.5 ± 22.4).

**Table A3.** GLMM results for effects of selection treatment and ambient environment on the ratio of emerged adult offspring to eggs laid.

| **Hypoxia experiment** | **Est** | **SE** | ***t*-value** | ***p*-value** |
| --- | --- | --- | --- | --- |
| Intercept | -0.255 | 0.026 | -9.787 | < 0.0001 |
| Selection treatment (hypoxia) | 0.018 | 0.036 | 0.505 | 0.61 |
| Ambient environment (hypoxia) | -0.069 | 0.037 | -1.863 | 0.063 |
| Treatment x environment | 0.043 | 0.054 | 0.787 | 0.43 |
| **Hypercapnia experiment** | **Est** | **SE** | ***t*-value** | ***p*-value** |
| Intercept | -0.233 | 0.029 | -7.934 | < 0.0001 |
| Selection treatment (hypercapnia) | -0.057 | 0.042 | -1.364 | 0.17 |
| Ambient environment (hypercapnia) | -0.022 | 0.035 | -0.632 | 0.53 |
| Treatment x environment | -0.027 | 0.051 | -0.532 | 0.56 |

**Table A4.** LMM results for effects of selection selection condition (control vs. selection treatment and ambient environment) on total adult offspring number.

| **Hypoxia experiment** | **Est** | **SE** | ***t*-value** | ***p*-value** |
| --- | --- | --- | --- | --- |
| Intercept | 72.497 | 3.969 | 18.267 | < 0.0001 |
| Selection condition (hypoxia) | -13.196 | 5.646 | -2.337 | 0.059 |
| **Hypercapnia experiment** | **Est** | **SE** | ***t*-value** | ***p*-value** |
| Intercept | 78.461 | 3.937 | 19.93 | < 0.0001 |
| Selection condition (hypercapnia) | -9.313 | 5.76 | -1.617 | 0.152 |

*Response to selection/adaptation*

Selection treatment had no effect on time to incapacitation or to recovery in the hypercapnia or hypoxia experiments. However, the effects of sex and of the interaction between sex and selection treatment were significant in all four models (Table A5, Figures A1­–4; Figures A5–8 show results by line). Time to incapacitation was longer in hypercapnia-selected than in control females (no change in males); and longer in hypoxia-selected than in control males (no change in females). Time to recovery was shorter in hypercapnia-selected than in control males (no change in females); longer in hypoxia-selected than in control males; and shorter in hypoxia-selected than in control females.

Selection under both hypoxia and hypercapnia decreased body size in both sexes (males: *F*_2,30_ = 133.7, *p* < 1e-14; females: *F*_2,30_ = 79.3, *p* < 1e-11; Tukey’s HSD *p*-values for control vs. selection all < 1e-6; Table A6, Figure A9).

**Table A5.** ANOVA results for effects of selection treatment and sex on resistance to CO_2_ knockout in the hypoxia and hypercapnia experiment lines.

| **Time to incapacitation** | | **SS** | **df 1** | **df 2** | ***F*** | ***p*** |
| --- | --- | --- | --- | --- | --- | --- |
| Hypercapnia | Selection treatment | 221 | 1 | 6 | 0.256 | 0.63 |
|  | Sex | 8009 | 1 | 150 | 9.293 | 0.003 |
|  | Treatment x sex | 6250 | 1 | 150 | 7.252 | 0.008 |
| Hypoxia | Selection treatment | 234 | 1 | 6 | 0.406 | 0.55 |
|  | Sex | 9257 | 1 | 150 | 16.051 | < 0.0001 |
|  | Treatment x sex | 5581 | 1 | 150 | 9.678 | 0.002 |
| **Time to recovery** | | **SS** | **df 1** | **df 2** | ***F*** | ***p*** |
| Hypercapnia | Selection treatment | 1981 | 1 | 6 | 0.490 | 0.51 |
|  | Sex | 244922 | 1 | 150 | 60.570 | < 0.0001 |
|  | Treatment x sex | 36663 | 1 | 150 | 9.067 | 0.003 |
| Hypoxia | Selection treatment | 640 | 1 | 6 | 0.148 | 0.71 |
|  | Sex | 20160 | 1 | 150 | 4.671 | 0.032 |
|  | Treatment x sex | 105576 | 1 | 150 | 24.462 | < 0.0001 |

**Table A6.** Summary statistics for effects of selection treatment on body size (wing length in mm; mean ± SD, n = 10 per group).

| **Sex** | **Treatment** | **Wing length** |
| --- | --- | --- |
| Males | Control | 1.335 ± 0.023 |
|  | Hypoxia | 1.108 ± 0.043 |
|  | Hypercapnia | 1.228 ± 0.023 |
| Females | Control | 1.503 ± 0.045 |
|  | Hypoxia | 1.258 ± 0.047 |
|  | Hypercapnia | 1.394 ± 0.035 |

**Supplementary Figures**

**Figure A1**

P1 results by line for the four hypoxia-selected and four corresponding control lines.

**Figure A2**

P2 results by line for the four hypoxia-selected and four corresponding control lines.

**Figure A3**

P1 results by line for the four hypercapnia-selected and four corresponding control lines.

**Figure A4**

P2 results by line for the four hypercapnia-selected and four corresponding control lines.

**Figure A5**

Time to incapacitation under CO_2_ anaesthetization for males and females in the hypercapnia-selected, hypoxia-selected, and respective control lines (n = 40 per group). Black line = mean, white box = 95% CI.

**Figure A6**

Time to incapacitation for males and females in each of the sixteen lines (n = 10 per group). For each of the two experiments, C1–4 = control lines, S1–4 = selection lines.

**Figure A7**

Time to recovery after CO_2_ anaesthetization for males and females in the hypercapnia-selected, hypoxia-selected, and respective control lines (n = 40 per group). Black line = mean, white box = 95% CI.

**Figure A8**

Time to recovery for males and females in each of the sixteen lines (﻿n = 10 per group). For each of the two experiments, C1–4 = control lines, S1–4 = selection lines.

**Figure A9**

Wing length (mm) for males and females in one line of each of the three selection treatments. Black line = mean, white box = 95% CI.

**Supplementary References**

Starmer, W. & Wolf, L.L. 1989. Causes of variation in wing loading among *Drosophila* species. *Biol. J. Linn. Soc.* **37**: 247–261.
